# Supplementary material for: A Humanized Yeast Phenomic Model of Deoxycytidine Kinase to Predict Genetic Buffering of Nucleoside Analog Cytotoxicity
Source: Genes (Basel). 2019 Sep 30;10(10):770. doi: 10.3390/genes10100770 (PMC6826991; doi:10.3390/genes10100770)
Supplement: Supplementary file 1 [file genes-10-00770-s001.zip › Additional_Files/Additional_File7_GOTermHeatmaps.zip!/Additional_File7_GOTermHeatmaps/termination_of_RNA_polymerase_II_transcription.pdf]

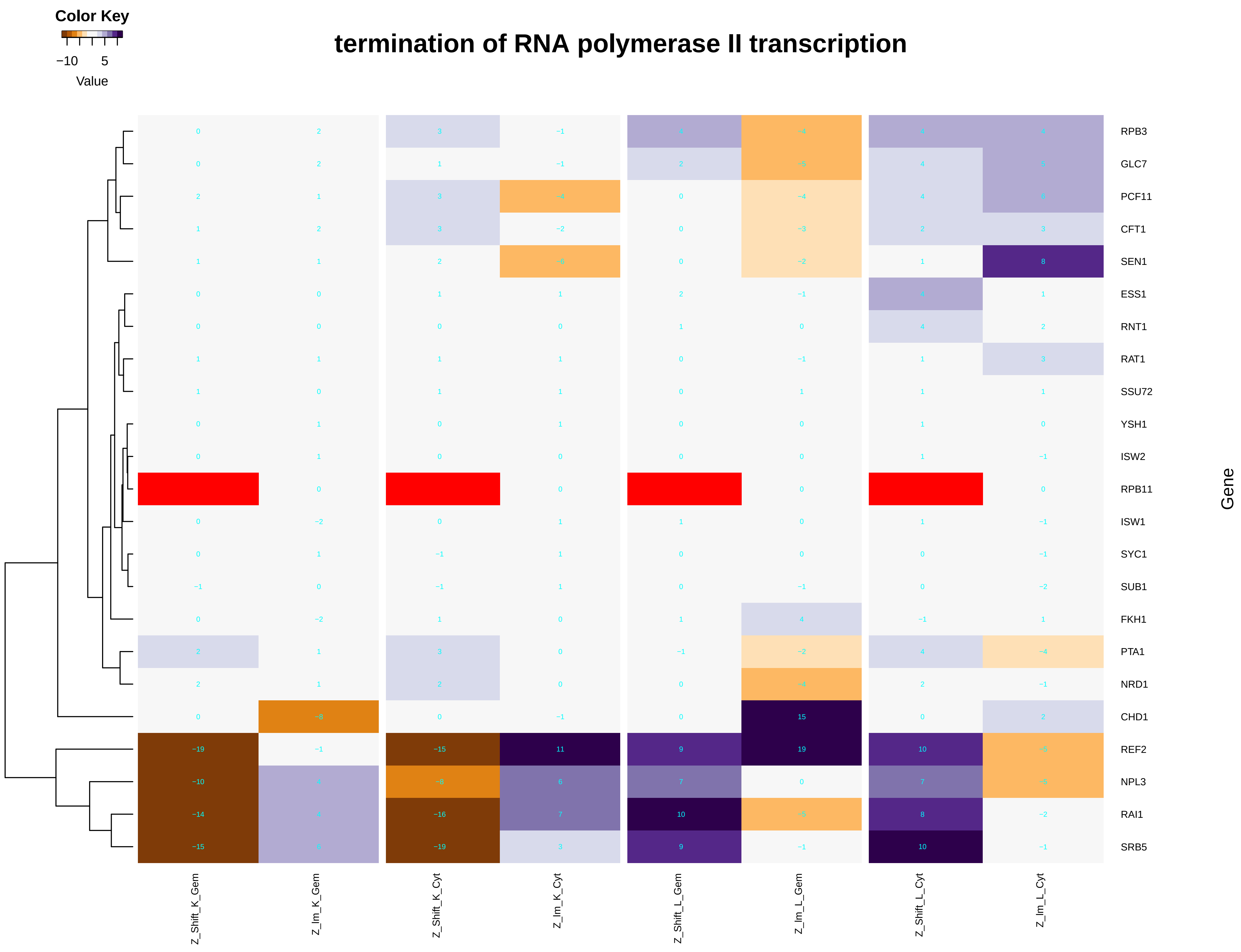

Color Key
−10
5
Value
termination of RNA polymerase II transcription
RPB3
GLC7
PCF11
CFT1
SEN1
ESS1
RNT1
RAT1
SSU72
YSH1
ISW2
RPB11
ISW1
SYC1
SUB1
FKH1
PTA1
NRD1
CHD1
REF2
NPL3
RAI1
SRB5
Gene
Z_Shift_K_Gem
Z_Im_K_Gem
Z_Shift_K_Cyt
Z_Im_K_Cyt
Z_Shift_L_Gem
Z_Im_L_Gem
Z_Shift_L_Cyt
Z_Im_L_Cyt

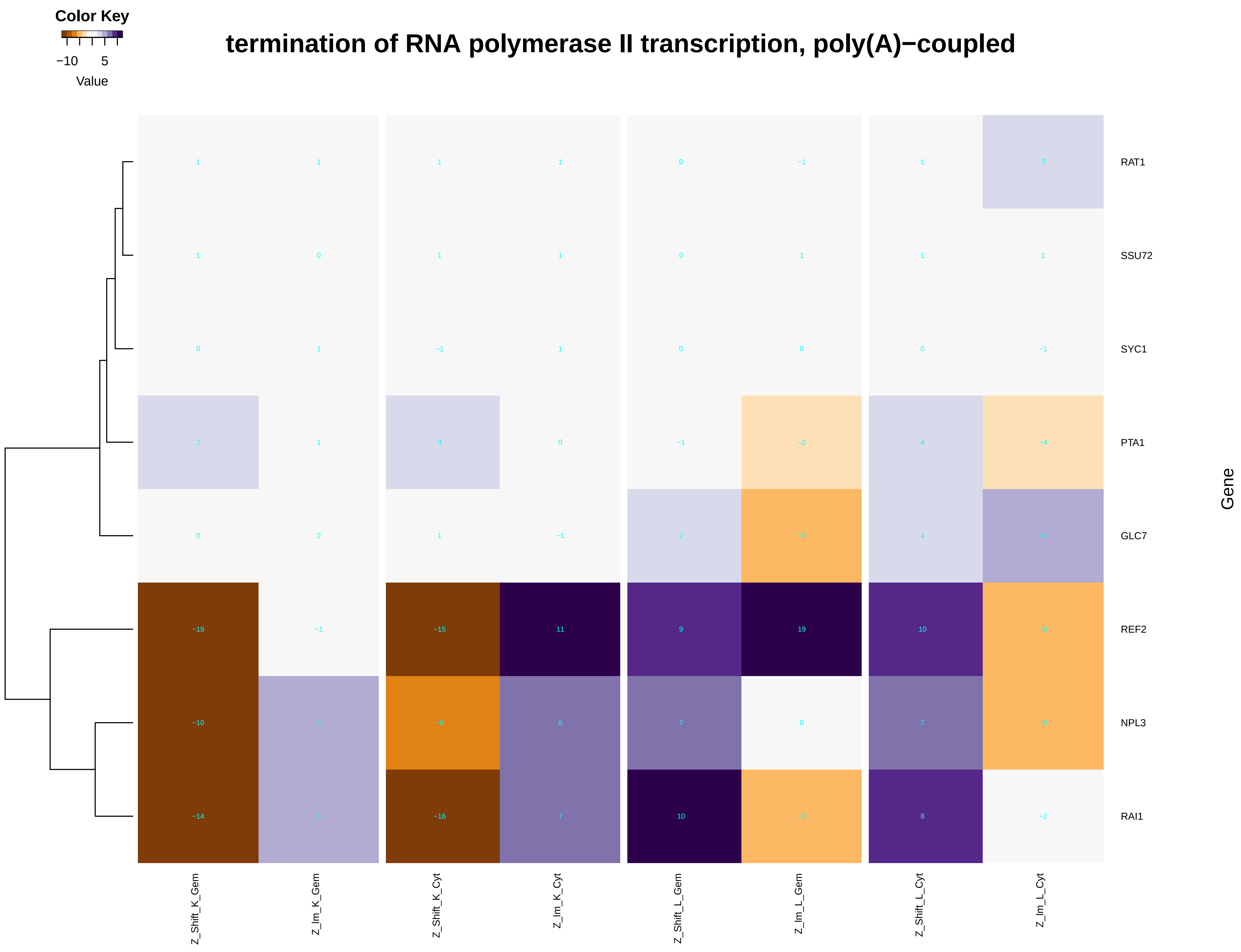
Color Key
−10
5
Value
termination of RNA polymerase II transcription, poly(A)−coupled
RAT1
SSU72
SYC1
PTA1
GLC7
REF2
NPL3
RAI1
Gene
Z_Shift_K_Gem
Z_Im_K_Gem
Z_Shift_K_Cyt
Z_Im_K_Cyt
Z_Shift_L_Gem
Z_Im_L_Gem
Z_Shift_L_Cyt
Z_Im_L_Cyt

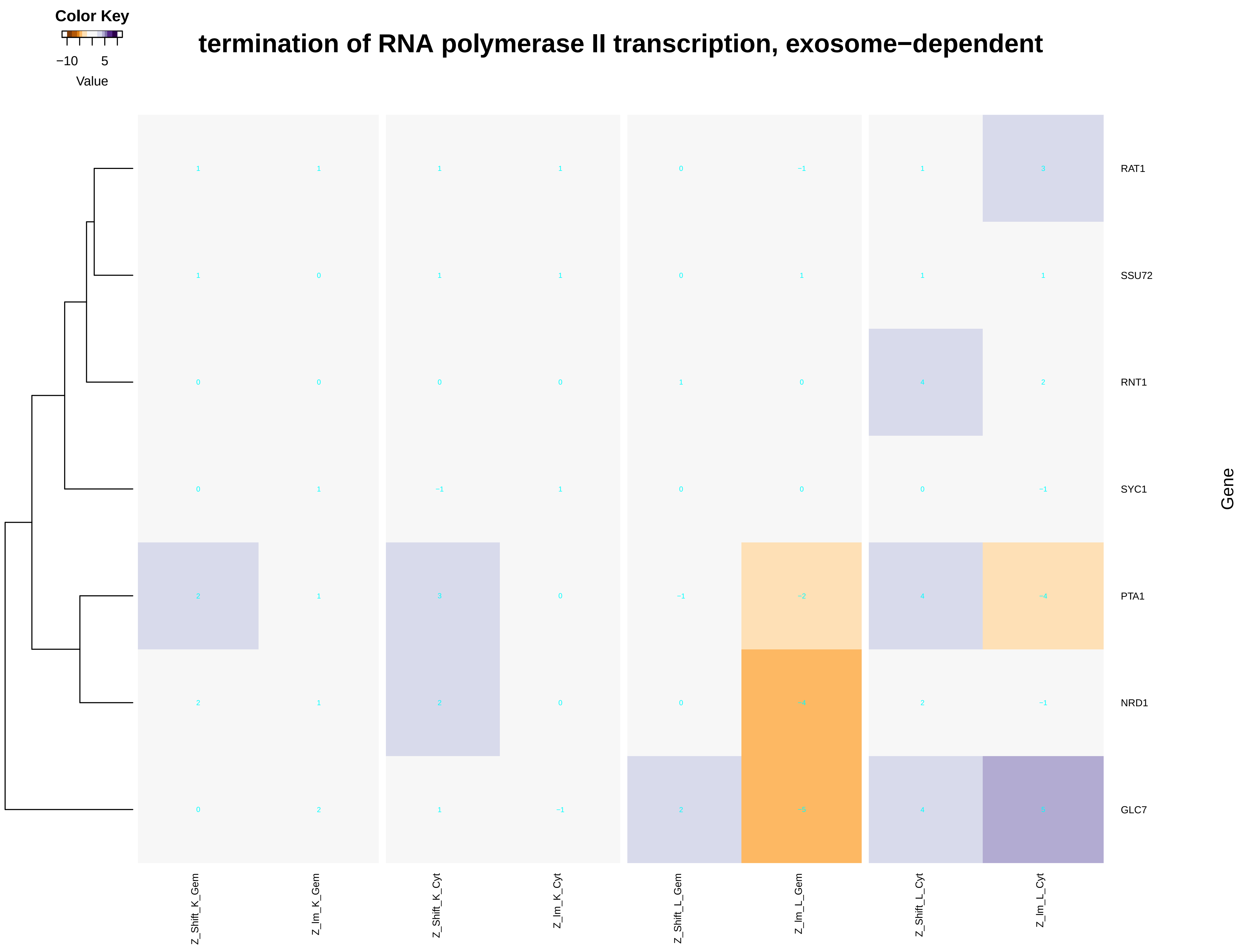

Color Key
−10
5
Value
termination of RNA polymerase II transcription, exosome−dependent
RAT1
SSU72
RNT1
SYC1
PTA1
NRD1
GLC7
Gene
Z_Shift_K_Gem
Z_Im_K_Gem
Z_Shift_K_Cyt
Z_Im_K_Cyt
Z_Shift_L_Gem
Z_Im_L_Gem
Z_Shift_L_Cyt
Z_Im_L_Cyt
